# Fecal Calprotectin Concentrations in Healthy Children Aged 1-18 Months

**DOI:** 10.1371/journal.pone.0119574

**Published:** 2015-03-05

**Authors:** Feng Li, Jingqiu Ma, Shanshan Geng, Junli Wang, Jinrong Liu, Jie Zhang, Xiaoyang Sheng

**Affiliations:** Department of Children and Adolescents Health Care, MOE-Shanghai Key Laboratory of Children’s Environmental Health, Xin Hua Hospital, Shanghai Jiao Tong University School of Medicine, Shanghai, China

## Abstract

**Objective:**

Fecal calprotectin (FC) is an established biomarker of gut inflammation. The aim of this study was to evaluate FC concentrations in healthy children between 1 and 18 months of age.

**Methods:**

Healthy children aged 1-18 months were enrolled in this study at the Department of Children's Health Care in Shanghai, China. Children’s stool samples were collected and analyzed, and FC concentration was determined using a commercially available enzyme-linked immunosorbent assay (ELISA). The children's weights and lengths were measured. Parents were asked to complete a brief questionnaire regarding several clinical and sociodemographic factors.

**Results:**

The FC concentrations were unevenly distributed; the median FC concentration was 174.3 μg/g (range: 6.0-1097.7 μg/g) or 2.241 log10 μg/g (range: 0.775-3.041 log10 μg/g) for all 288 children. The children were divided into several age groups: 1-3 months, 3-6 months, 6-9 months, 9-12 months and 12-18 months. The median FC concentrations for these age groups were 375.2 μg/g (2.574 log10 μg/g), 217.9 μg/g (2.338 log10 μg/g), 127.7 μg/g (2.106 log10 μg/g), 96.1 μg/g (1.983 log10 μg/g) and 104.2 μg/g (2.016 log10 μg/g), respectively. A significant correlation between age and FC concentration was found (*r*=-0.490, *p*<0.001). A simple correlation analysis of weight-for-length Z-scores or weight-for-age Z-scores vs. FC concentrations showed that these variables were negatively correlated (Spearman’s rho=-0.287, *p*<0.001; Spearman’s rho=-0.243,* p*<0.001, respectively).

**Conclusions:**

The FC levels of children aged 1-18 months exhibit a downward trend with increasing age and are greater than the normal levels observed in healthy adults. In healthy children aged <6 months, FC levels are high. In children aged 6-18 months, FC concentrations are relatively low but are still higher than those of children aged >4 years.

## Introduction

Calprotectin is a calcium- and zinc-binding protein and a member of the S100 family of proteins. It has a molecular mass of 36.5 kDa [1], and it has both bactericidal and fungicidal properties [2]. Calprotectin is secreted extracellularly from stimulated neutrophils [3], eosinophils [4] and monocytes [5] and is expressed in some mucosal epithelial cells [6, 7]; it is also released during cell disruption and death [8]. When bound to calcium, calprotectin has a high heat resistance and is stable in stool samples for up to one week at room temperature [9, 10]. These properties allow calprotectin to be eliminated intact in the feces and give it an advantage as a noninvasive biochemical marker for the screening of intestinal inflammation, compared with other markers that are currently used (lactoferrin, neutrophil elastase, and leukocyte esterase)[11]. Fecal calprotectin (FC) most likely originates from the transepithelial migration of granulocytes (neutrophils and eosinophils) [4, 12], and FC levels are quantitatively related to granulocytes migration towards the gastrointestinal tract [13–15]; this migration correlates with endoscopic and histological activity scores in inflammatory bowel diseases [16, 17]. Thus, FC, in particular, has long been regarded as a promising marker of gastrointestinal pathology [18] and a reliable and valuable marker for the detection of gastrointestinal tract inflammatory activity [9, 19–21] and cystic fibrosis [22], and FC measurements can be used to screen for intestinal disease [23].

Despite the large volume of literature on calprotectin in recent years, insufficient data on FC in children are available; most studies of FC in children have been case-control studies that included healthy children as controls, and these had a variety of limitations, including small sample sizes and an undefined reference range for healthy children. There are conflicting data concerning appropriate reference values in children [24–34]. Despite the small sample sizes of these studies, because of the disparities in the presentation of the data (means, together with standard deviations, standard errors of the mean, or 95% confidence intervals, in some studies and medians and ranges in others), pooling the data in these studies is not practical[35]. Thus, Sykora et al. have suggested that further studies investigating FC reference ranges are required to allow routine measurements to be made, especially in subjects younger than 12 months of age [21]. Further research is needed to study the normal development of FC levels in infants and the functional importance of FC in infancy [26]. Reference values for FC in children have not been defined for many populations worldwide, and in particular, reference values for FC in Chinese children are not known. Thus, the aim of our study was to describe the concentrations of FC in healthy full-term children aged 1–18 months.

## Subjects and Methods

### Subjects

This study was performed at the Department of Children and Adolescent’s Health Care of Xinhua Hospital, which is affiliated with the Shanghai Jiaotong University School of Medicine; the study participants received routine physical examination at the Department of Children and Adolescent’s Health Care of Xinhua Hospital. The subjects, aged 1–18 months, were consecutively recruited from April 2013 to March 2014. At enrollment, the parents of the children were asked to complete a brief health questionnaire regarding several clinical and sociodemographic factors. Clinical features, including gestational age, birth weight, sex, Apgar score, postnatal age, neonatal diseases, symptoms, physical examination findings, weight and length, were recorded prior to the collection of each stool sample. All children recruited to participate in this study met the following inclusion criteria: age 1 month to 18 months; gestational age >37 completed weeks; 5-minute Apgar score >7; birth weight appropriate for gestational age (2,500–4,000 g); and no illnesses in the month prior to enrollment, and thus no known underlying chronic inflammatory disease. The exclusion criteria were the following: any intake of steroidal or non-steroidal anti-inflammatory drugs, gastric acidity inhibitors, antibiotics or any other drug during the 2 weeks prior to recruitment; nasal bleeding during the final week before the study; or a history of signs or symptoms of infection or gastrointestinal disease (diarrhea, vomiting, hematochezia, fever).

A total of 315 children were invited to participate in the study; 15 (4.8%) families declined to participate, and 12 (3.8%) potential subjects were excluded due to vomiting (3), acute upper respiratory tract infection (3), incomplete data (2), nasal bleeding (1) or other medical conditions (3). Ultimately, 288 healthy, eligible children were included in the final analysis. We categorized the children into six age groups: 1–3 months, 3–6 months, 6–9 months, 9–12 months and 12–18 months. Fecal samples were obtained from these 288 healthy children.

### Anthropometric measurements and calculations

The children’s weights and supine lengths were measured using standard techniques. Anthropometric measurements of the infants were performed in duplicate by a trained member of the research team, as described in our previous study [29]. Length-for-age Z-scores (LAZ), weight-for-age Z-scores (WAZ), and weight-for-length Z-scores (WLZ) were calculated using Anthro software (version 3.1) based on the World Health Organization Child Growth Standards.

### Stool collection

The parent of each child was provided with a plastic container and was instructed on how to collect stool samples. Parents removed the fecal sample from their child’s diaper, and the sample was brought or sent in screw-capped containers to the hospital. All fecal samples were frozen and stored at-80°C immediately following receipt until analysis.

### Fecal calprotectin measurements

The calprotectin concentration in each sample was determined using a commercially available enzyme-linked immunosorbent assay (ELISA) that quantitatively measures calprotectin levels (Bühlmann Laboratories AG, Schönenbuch, Switzerland), as previously described in our study[29] and another study[36]. The total coefficient of variation for this analysis was 15%, including the extraction procedure, with intra- and inter-assay coefficients of variation of 4.7% and 4.1%, respectively [36]. Included in each sample run were blanks, standards and controls. Prior to analysis, frozen stool samples were thawed at room temperature. If the sample yielded a reading greater than the maximum calibrated level (600 μg/g), the remaining extract of the sample was further diluted 1:6 with incubation buffer, and the assay was repeated. Calprotectin levels are expressed as μg/g of feces.

### Ethical considerations

The study was approved by the Institutional Ethics Committee of Xinhua Hospital (XHEC-D-2012–015) and conducted in accordance with the revised Declaration of Helsinki. Written, informed consent was obtained from the parents of all of the children who participated in this study prior to their enrollment.

### Statistical analyses

Statistical analyses were performed using SPSS version 17.0 for Windows (SPSS Inc., Chicago, IL, USA). LAZ, WAZ and WLZ were compared using t-tests. FC concentrations are presented as median, percentages and range. FC concentrations were also transformed to their base 10 logarithm (log10). FC values in the different age groups were compared using the Kruskal-Wallis and Mann-Whitney tests. Differences between variables were considered statistically significant when *p*<0.05. A simple regression analysis was performed to assess the correlation between FC concentration and age. Spearman’s correlation test was used to evaluate the relationship between selected variables and FC values.

## Results

**Fig 1:**
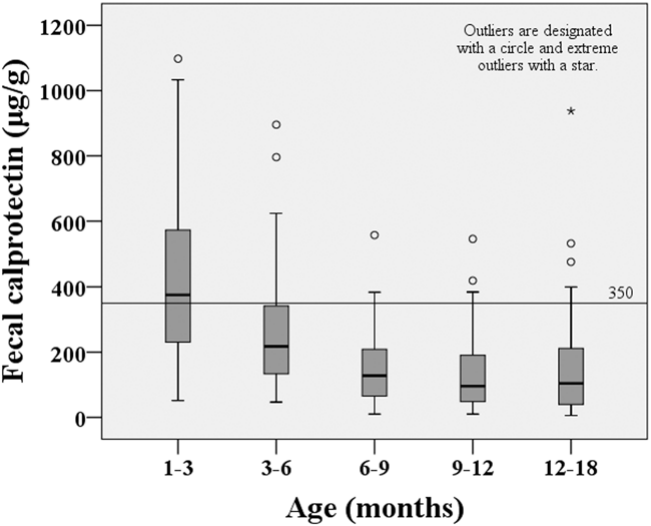
Fecal calprotectin concentrations in six age groups of healthy children.

The study population included 288 children (115 females and 173 males) ranging in age from 1 to 18 months. The subjects were born at a median gestational age of 39 weeks (range 37–42 weeks), with a median birth weight of 3302 g (range 2500–4000 g). The numbers of children in each of the 5 age groups were 75 (1–3 months), 61 (3–6 months), 51 (6–9 months), 51 (9–12 months) and 50 (12–18 months). The mean values of LAZ, WAZ and WLZ were similar in all of the subgroups (*p*>0.05; Table 1). The FC concentrations were unevenly distributed, with a median FC concentration of 174.3 μg/g [range: 6.0–1097.7 μg/g; interquartile range (IQR): 76.3–333.6 μg/g] or 2.241 log10 μg/g (range: 0.775–3.041 log10 μg/g; IQR: 1.882–2.523 log10 μg/g) in all the subjects aged 1–18 months. The median values in the 5 age groups were 375.2 μg/g (1–3 months, range: 51.8–1097.7 μg/g, IQR: 229.0–575.1 μg/g; median: 2.574 log10 μg/g, range: 1.714–3.041 log10 μg/g, IQR: 2.360–2.760 log10 μg/g), 217.9 μg/g (3–6 months, range: 47.0–895.4 μg/g, IQR: 124.0–324.7 μg/g; median: 2.338 log10 μg/g, range: 1.672–2.952 log10 μg/g, IQR: 2.092–2.535 log10 μg/g), 127.7 μg/g (6–9 months, range: 10.0–557.2 μg/g, IQR: 65.1–214.3 μg/g; median: 2.106 log10 μg/g, range: 1.000–2.746 log10 μg/g, IQR: 1.814–2.331 log10 μg/g), 96.1 μg/g (9–12 months, range: 10.0–545.4 μg/g, IQR: 47.5–200.7 μg/g; median: 1.983 log10 μg/g, range: 1.000–2.737 log10 μg/g, IQR: 1.677–2.303 log10 μg/g) and 104.2 μg/g (12–18 months, range: 6.0–937.5 μg/g, IQR: 39.9–211.9 μg/g; median: 2.016 log10 μg/g, range: 0.775–2.972 log10 μg/g, IQR: 1.600–2.326 log10 μg/g). These results are shown in Table 1 and Fig. 1. In each age group, no difference in FC concentration was observed between genders (median FC, males: 154.3 μg/g, females: 212.4 μg/g; *p* = 0.087). Males and females also had similar calprotectin concentrations in all of the subgroups (*p*>0.05). Among the subjects aged <12 months (238 children), 90 children (38%) were breastfed, and 148 children (62%) were not breastfed. The median FC concentration was significantly higher in breastfed children (323.5 μg/g) than in formula-fed children (148.3 μg/g, *p*<0.001).

**Table 1:** Fecal calprotectin concentrations, LAZ, WAZ and WLZ in apparently healthy children.

Age (months)	Boys		Girls		All					
	N	Median FC (5th–95th), μg/g	N	Median FC (5th–95th), μg/g	N (%)	Median FC (5th–95th),μg/g	Median FC (5th–95th), log10μg/g	LAZ (Mean±SD)	WAZ (Mean±SD)	WLZ (Mean± SD)
1–3	45	323.2(78–867)	29	378.1(70–1065)	75(26.0)	375.2(77–962)	2.574(1.885–2.983)	0.505±1.158	0.694±0.132	0.477±0.891
3–6	35	217.9(50–635)	26	219.7(54–800)	61(21.2)	217.9(53–621)	2.338(1.726–2.793)	0.671±1.003	0.777±0.051	0.511±0.913
6–9	31	127.7(10–453)	20	123.5(35–338)	51(17.7)	127.7(10–362)*	2.106(1.000–2.558)	0.798±0.737	0.844±0.782	0.575±0.958
9–12	29	94.8(10–482)	22	109.5(14–382)	51(17.7)	96.1(10–398)*	1.983(1.000–2.600)	0.825±0.713	0.918±0.831	0.737±1.090
12–18	32	89.8(17–386)	18	177.9(20–532)	50(17.4)	104.2(10–501)*	2.016(0.998–2.670)	0.600±0.721	0.708±0.820	0.568±1.034
All	173	154.3(18–623)	115	212.4(34–904)	288	174.3(24–764)	2.241(1.370–2.883)	0.665±0.918	0.780±0.954	0.563±0.968

*Difference in median,

*p*<0.05 compared with children younger than 6 months of age;

5th–95th: 5th–95th percentile,

FC: fecal calprotectin, LAZ: length-for-age Z-score, WAZ: weight-for-age Z-score, WLZ: weight-for-length Z-score.

When the five age groups were analyzed simultaneously, a significant between-group difference was found (*p*<0.001). Fecal calprotectin levels were significantly higher in healthy children aged 1–3 months than in healthy children aged 3–6 months (375.2 μg/g vs. 217.9 μg/g, *p*<0.001). Younger children (1–6 months) had substantially higher levels of FC (median: 282.7 μg/g; range: 47.0–545.4 μg/g; IQR: 156.3–483.1 μg/g) compared to healthy older children (6–18 months, median: 114.9 μg/g; range: 6.0–937.5 μg/g; IQR: 54.9–203.0 μg/g; *p*<0.001). No statistically significant difference in FC level was found among the children aged 6–18 months (6–9 months vs. 9–12 months, 6–9 months vs. 12–18 months and 9–12 months vs. 12–18 months, *p*>0.05). We found that the younger children exhibited a trend towards higher concentrations of FC (Table 1 and Fig. 1), and a statistically significant correlation was found between age and FC concentration (Spearman’s rho = -0.490, *p*< 0.001).

**Fig 2:**
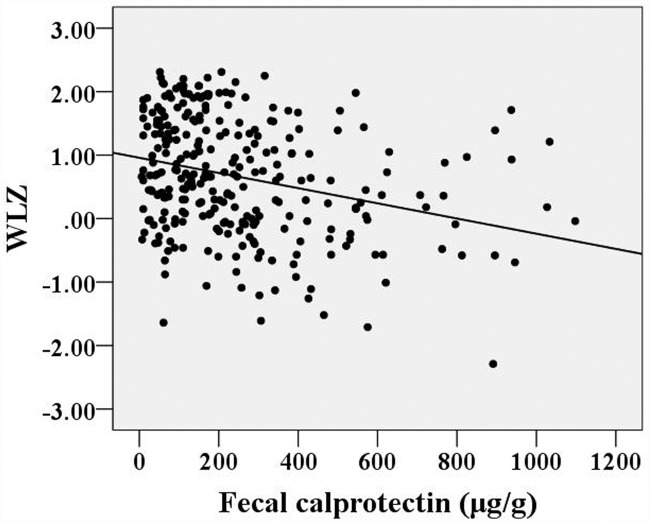
Correlations between weight-for-length Z-scores and fecal calprotectin concentrations.

**Fig 3:**
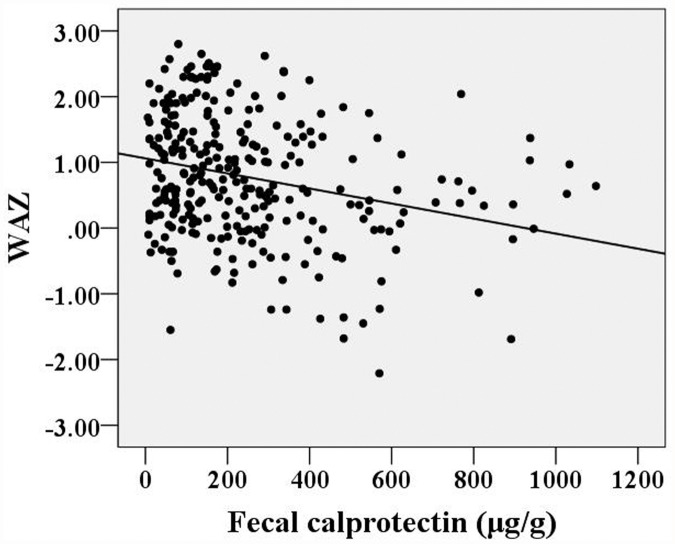
Correlations between weight-for-age Z-scores and fecal calprotectin concentrations.

Twenty-seven percent of the subjects (78 cases) and 25% of the subjects aged <12 months (59 cases) had an FC concentration >350 μg/g, which has been reported as an upper limit of a normal range during the first year of life [37]. A simple correlation analysis of WLZ or WAZ with FC showed that these variables were negatively correlated (Spearman’s rho = -0.287, *p*<0.001; and Spearman’s rho = -0.243, *p*<0.001, respectively; Figs. 2 and 3).

## Discussion

This is the first survey of FC concentrations in an apparently healthy population aged 1 to 18 months in China. In our study, the median FC concentration was 174.3 μg/g in 288 healthy children. Higher values have previously been found in infants compared to older children [20, 24, 30, 38]. Consistent with previous results [25, 27, 39], our data confirm that infants in the first months of life have higher calprotectin concentrations than do healthy older children; the median values were 375.2 μg/g (1–3 months) and 217.9 μg/g (3–6 months) in the younger infants, after which the values dropped to approximately 100 μg/g (6–18 months) in the older infants and toddlers. Thus, the FC levels of 1–18-month-old children showed a downward trend with age. In our study, we provided the range of measured FC concentrations for subjects aged less than 18 months; these data could be of use in clinical practice as reference ranges for FC levels in children in between 1 and 18 months of age.

In the present study, FC levels varied with age and showed a significant and negative correlation with age. Children in the first months of life have high calprotectin levels; several underlying mechanisms and possible explanations [21] for this finding exist. First, FC levels may be high in the first months of life due to ongoing developmental processes occurring in the digestive tract during this stage of life. A high calprotectin concentration in feces appears to be a normal phenomenon in infants and may be related to the immaturity of adaptive immunity in infancy [26]. The high levels of FC in infants may reflect increased transepithelial migration of either granulocytes or newly recruited macrophages, and this migration could be related to intestinal mucosal immaturity [25, 40]. A second reason why FC levels may be high in the months after birth is that intestinal epithelial barrier function is immature during this time, and infants’ bodies have not developed the ability to regulate the microbial flora in the gut. High FC levels have been identified in healthy neonates in previous studies, and the findings of these studies have suggested that intestinal permeability is increased during the first weeks of life [25, 27, 41]. Because calprotectin is primarily derived from granulocytes, its concentration is directly proportional to the extent of granulocyte migration to the intestinal tract [14]. Thus, these high basal calprotectin levels could be due to higher intestinal permeability, the establishment of gut flora, and a response to alimentary antigens, as well as to the colonization of the gut by commensal microbes, which help to prevent infections with enteric pathogens and block interactions between pathogens and host cells [31, 39, 42, 43]. It has been hypothesized that intestinal colonization during the first weeks of life and potent chemotactic agents (such as formyl-methionyl-leucyl-phenylalanine) have an important role in stimulating the transepithelial migration of granulocytes through the mucosal membrane during the development of oral tolerance and the regulation of the microbial flora[9], leading to a higher FC concentrations[44]. A third reason why FC concentrations may be high in the months after birth is that subclinical physiological inflammation may be present in the digestive tract, and such inflammation could result in the migration of granulocytes into the gut lumen [15, 21, 32]. Because calprotectin exhibits many biological activities, including bactericidal and fungicidal activities and immunomodulatory properties, it may be postulated that this protein could exert a beneficial effect on host defense in physiologically normal environments such as the intestinal ecosystem in healthy children during the first weeks of life [39]. We found that the type of feeding influences the FC concentration and that breastfed infants (<12 months) have higher FC levels than non-breastfed ones in the first months of life. This may reflect the fact that immunomodulatory factors in human milk influence the gut mucosa, as we described previously [45]. In the present study, we found that FC and anthropometric indices were negatively correlated, consistent with the findings of a previous study that age-corrected growth in both weight and height was negatively related to intestinal permeability (*r* = -0.41, *p*<0.001), i.e., that growth was poorer with higher permeability values[46]. Thus, children exhibiting poor growth may have higher intestinal permeability, leading to a higher FC concentration. In this study, the wide range of inter- and intra-individual variation in calprotectin excretion in feces may also be explained by normal biological variability, with day-to-day variations as have been described previously in adults and children [30, 47, 48].

Simple and noninvasive tests are needed in pediatric care. The Bühlmann calprotectin assay (Bühlmann Laboratories AG, Schönenbuch, Switzerland) yields the following threshold values for adults and children 4 to 17 years of age: normal values, FC values <50 μg/g; mild organic disease, FC values between 50 and 200 μg/g; and active organic disease, FC values >200 μg/g. However, these threshold values are not applicable to children 1 to 18 months of age. A large volume of literature concerning normal FC values in children exists. Olafsdottir et al. [26] found that mean FC levels were significantly higher in healthy infants than in healthy children aged >1 year (278 μg/g vs. 40 μg/g). Hestvik et al. [33] analyzed FC levels in healthy children and found that the median FC concentrations were 249 μg/g in children <1 year old, 75 μg/g in 1–4-year-olds and 28 μg/g in 4–12-year-olds. Berni et al. [49] reported that the median FC value was 28 μg/g in healthy children (age range 13–216 months), and Ezri et al. suggested that normal values vary with age, with higher cut-off values for children during the first year of life (<350 μg/g) than for older children (<275 μg/g) or adults (<50 μg/g) [37]. Although FC levels are considered to vary with age, 50 μg/g is considered to be useful a cut-off for individuals more than 4 years old [30, 33, 50, 51]. While 50 μg/g has thus been proposed as a cutoff that can be used for screening purposes, different cut-off values have been suggested for different patient groups. Some studies have investigated the measurement of FC levels as a diagnostic test in children. In a meta-analysis of prospective studies performed by von Roon et al., which included a total of 5,983 patients, the pooled sensitivities and specificities of FC levels in differentiating inflammatory bowel disease (IBD) from non-IBD diagnoses were 86% and 81%, respectively, with the higher precision at a cut-off of 100 μg/g[52]. FC may also be a useful marker for necrotizing enterocolitis (NEC), as an FC value of 792 μg/g was found to be 76% sensitive and 92% specific for the diagnosis of NEC [53]. The optimal cut-off value for FC to discriminate between neonates with NEC and those with suspected NEC and alternative final diagnoses was 286.2 μg/g, which resulted in a specificity of 93% and sensitivity of 86% for this test [54]. Moreover, it has been found that FC levels decrease as NEC resolves [55]. However, the usefulness of FC as a marker of disease may be controversial because high inter-individual variations occur in healthy infants [55]. In the current study, the observed FC levels in healthy children 0 to 6 months of age (median: 282 μg/g) were higher than those found in some previous studies [25, 27, 28, 32] and similar to those reported in other studies [24, 26, 33]; the differences in FC levels in across these studies may be influenced by genetic and environmental factors or a combination of both [56]. Due to inter-individual variability, sequential measurements of calprotectin should also be considered as an alternative to using defined cut-off levels; it would also be useful to investigate the utility of sequential FC measurements in a prospective study [51]. Fecal calprotectin levels should be evaluated carefully in younger children, especially during the first year of life [57]. Kapel et al. reviewed data on 1180 term and preterm neonates examined in twenty studies and found that an FC value greater than approximately 350 μg/g may indicate the presence of a digestive disease, when 350 μg/g was used as the upper reference limit, as recommended in the literature [39]. In our study, 27% (78) of children had an FC concentration greater than this upper limit; however, they displayed no symptoms of intestinal infection, such as vomiting, diarrhea, fever, or other specific symptoms, and the potential association between their high FC level and the presence of intestinal infection should be investigated further. FC levels should be investigated in larger cohorts of children (especially <12 months of age), and the periods surrounding intestinal development and digestive distress, in particular, should be analyzed.

### Limitations

Our study has several limitations. First, a potential weakness is that stool samples were collected from children’s diapers. Olafsdottir et al. [26] reported that this method of collection increases FC concentration by up to 30% because water is absorbed into the diaper. This could yield higher FC levels to be measured than are actually present, and therefore, direct stool collection at emission may be more practical[21]. Second, we did not further investigate the children with elevated FC concentrations to evaluate whether these concentrations normalized over time. Therefore, we are unable to provide an explanation for the FC levels that were higher than 350 μg/g. Third, we did not determine the FC concentration in healthy children aged 1.5–4 years in China; we anticipate undertaking this task in future work.

## Conclusions

The FC levels of children aged 1–18 months show a downward trend with age and are greater than the normal levels observed in healthy adults and older children. In healthy children of age <6 months, FC levels are high. In children aged 6 to 18 months, FC concentrations are lower than those of children of age <6 months, but they are still higher than those of children of age >4 years. The ranges of FC values found in this study could be of use in future studies exploring the feasibility of using FC measurements to investigate gastrointestinal disorders in children. The possible relationship between FC levels and the health of the intestinal mucosa in children (especially <12 months) should be studied further.
